# “Novel” Triggers of Herpesvirus Reactivation and Their Potential Health Relevance

**DOI:** 10.3389/fmicb.2018.03207

**Published:** 2019-01-07

**Authors:** Tobias Stoeger, Heiko Adler

**Affiliations:** ^1^Institute of Lung Biology and Disease, Comprehensive Pneumology Center, Helmholtz Zentrum München - German Research Center for Environmental Health (GmbH), Neuherberg, Germany; ^2^Research Unit Lung Repair and Regeneration, Comprehensive Pneumology Center, Helmholtz Zentrum München - German Research Center for Environmental Health (GmbH), and University Hospital Grosshadern, Ludwig-Maximilians-University, Munich, Germany; ^3^German Center for Lung Research (DZL), Giessen, Germany

**Keywords:** herpesvirus, reactivation, nanoparticle, non-canonical triggers, health relevance

After primary infection, herpesviruses persist for life in their hosts in a latent stage (Adler et al., 2017). Different subfamilies of herpesviruses establish latency in specific and different sets of cells (Pellett and Roizman, 2013; Lieberman, 2016). The latent stage can be interrupted by periods of lytic replication, termed reactivation. Reactivation is important for viral spread to new hosts or for the maintenance of the viral reservoir in the host. Usually, reactivation is not associated with disease but under certain circumstances, it may be accompanied by clinical symptoms. The stimuli and the precise molecular mechanisms that lead to reactivation from the latent state are not fully understood and can differ from one herpesvirus to another.

## Herpesvirus Reactivation in The Human Host by “Classical” Triggers

**Figure 1:**
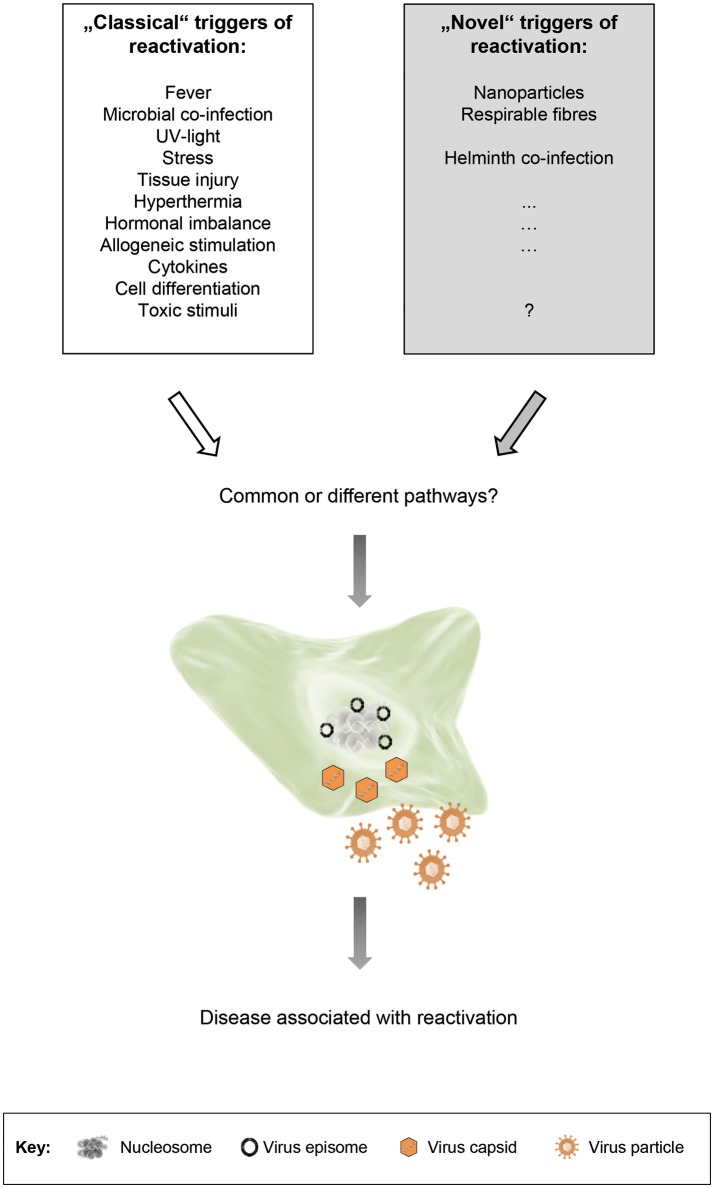
“Classical” and “novel” triggers of herpesvirus reactivation. Both types of triggers are able to induce reactivation of latent herpesviruses. The respective signal transduction pathways that are activated may be shared, or may vary depending on the stimulus, the cell type and the virus. Although reactivation is usually not associated with disease, it may be accompanied by clinical symptoms under certain circumstances.

A number of stimuli that trigger reactivation in humans are known for a long time – we term them as “classical” triggers of herpesvirus reactivation (Figure 1): (i) Alphaherpesviruses, e.g., latent HSV-1 in neurons of various ganglia, are for example reactivated by local injury to tissues innervated by latently infected neurons or by systemic physical or emotional stress, fever and microbial co-infection as well as UV-exposure or hormonal imbalance (Roizman and Whitley, 2013; Roizman et al., 2013). (ii) Reactivation of betaherpesviruses, for example CMV, is observed commonly in the setting of immunosuppression, particularly where allogeneic stimulation and pro-inflammatory cytokines are present and stimulate cellular differentiation to macrophages or dendritic cells (Stinski and Meier, 2007; Liu et al., 2013; Dupont and Reeves, 2016; Lieberman, 2016). (iii) Stimuli that induce reactivation of gammaherpesviruses, for example EBV, are differentiation of B cells into plasma cells through antigen stimulation of the B-cell receptor. *In vitro*, and potentially likewise *in vivo*, also cytokines including TGF-beta can induce B-cell activation and thus result in lytic EBV infection. Additionally, host cell stress, induced for example by chemotherapy or body irradiation, can reactivate latent EBV. In cell culture, EBV reactivation can also be triggered by phorbol ester 12-0-tetradecanoyl phorbol-13-acetate (TPA), sodium butyrate or calcium ionophores (Kenney, 2007; Murata, 2014). Many of these reactivation triggers activate classical signal transduction pathways, including protein kinase C, p38 kinase, c-Jun N-terminal kinase (JNK), ERK kinase and PI3 kinase. In conclusion, there are many settings of a specific herpesvirus, a specific host cell and a specific stimulus, determining the transition from latency to lytic cycle (Kenney, 2007; Murata, 2014; Dupont and Reeves, 2016; Cliffe and Wilson, 2017).

## “Novel” Triggers of Herpesvirus Reactivation

Beside the above described “classical” triggers of herpesvirus reactivation, we propose the existence of additional, so far unappreciated “novel” triggers of herpesvirus reactivation (Figure 1). This proposal is based on recent findings by us and by others. Reese et al. found that helminth co-infection reactivated murine gammaherpesvirus 68 (MHV-68) *in vivo* in an IL-4/Stat6-dependent manner (Reese et al., 2014; Reese, 2016). We demonstrated that cells persistently infected with murine or human gammaherpesviruses responded to nanoparticle (NP) exposure by reactivation of latent virus and by restoring a molecular signature found during productive infection (Sattler et al., 2017). In our study, we exposed cells or mice latently infected with MHV-68 to different NP. *In vitro*, NP-exposure resulted in expression of lytic viral genes and virus production. *In vivo*, an increase in lytic viral proteins and gene expression was observed in lungs and cells from bronchoalveolar lavage. The patterns of gene and metabolite expression in whole lung tissue were strikingly similar to acute virus infection. In human cells latently infected with EBV, NP-exposure also induced virus production. The carbonaceous NPs used in our study were (i) carbon black like surrogates of environmental NPs, derived for example from combustion or mass production, as well as (ii) carbon nanotubes (CNTs) as examples for promising new materials in technology and biomedicine. Thus, it is well-conceivable that other types of NPs, derived from different materials, might also be able to induce herpesvirus reactivation. Furthermore, a variety of additional factors, present in the environment but so far not considered to be triggers of herpesvirus reactivation, might be relevant too.

## Potential Health Relevance of “Novel” Inducers of Herpesvirus Reactivation

The finding, that helminth co-infection wakes up dormant gammaherpesviruses might have major implications for human health. This has already been discussed by Maizels and Gause (Maizels and Gause, 2014). Equally, our discovery that exposure to NP is able to activate gammaherpesviruses that are dormant in the lung may have consequences for human health in an environment with an increasing exposure to NP. Both innate and adaptive immune responses are modulated by NP, leading for example to immunosuppression or hypersensitivity (Pallardy et al., 2017). Exposure to ambient respirable particles such as man-made mineral fibers or vehicle exhaust emissions have been associated with various adverse health effects (Seaton et al., 2010). When inhaled, NP deposit efficiently and persistently in the alveolar region of the respiratory tract. Their pro-inflammatory properties shape chronic lung diseases like asthma, chronic obstructive pulmonary disease (COPD), pulmonary fibrosis or cancer (Byrne and Baugh, 2008; Morgenstern et al., 2008; Bonner, 2010; Sese et al., 2018; Siroux and Crestani, 2018). The rapid expansion of nanotechnology bears the risk of increasing the incidences of these diseases. Herpesvirus infections may also contribute to the development of chronic pulmonary diseases (Meneghin and Hogaboam, 2007; Vannella and Moore, 2008; Naik and Moore, 2010; Kropski et al., 2012). However, potentiation by a combined exposure to both triggers has not been investigated. We propose that repeated NP exposure and concomitant herpesvirus reactivation result in chronic inflammatory and remodeling processes in the lung, for example by permanently stimulating an aberrant immune response, finally leading to immunopathology and disease development. It is tempting to speculate that inhaled NP might also be among the unknown triggers suspected to enable propagation of additional resident viruses of the airway virome, thereby causing exacerbations of various lung diseases (Marsland and Gollwitzer, 2014).

Following exposure via various routes including inhalation, NP are not only found in the lung but also deposited in numerous organs including the central nervous system (Hong et al., 2017; You et al., 2018). Inhaled nanoparticles can translocate into the systemic circulation and have been shown to accumulate at sites of vascular inflammation and disease (Miller et al., 2017). There is a long term controversy with regard to the contribution of herpesviruses to other chronic diseases beyond the lung: For example, CMV has been associated with coronary heart disease (atherosclerosis) (Du et al., 2018), and EBV, HHV-6 and VZV with multiple sclerosis (Geginat et al., 2017). We propose that also in these disease entities, an interaction of NP with latent herpesviruses may result in reactivation with subsequent chronic inflammation and disease development.

Taken together, future studies on effects of NP-induced herpesvirus reactivation on human health, possible treatments and potential regulatory measures are warranted.

## Author Contributions

All authors listed have made a substantial, direct and intellectual contribution to the work, and approved it for publication.

### Conflict of Interest Statement

The authors declare that the research was conducted in the absence of any commercial or financial relationships that could be construed as a potential conflict of interest.
